# Observations on the Structure and Development of Ovarian Teeth

**Published:** 1890-07

**Authors:** T. Charters White, J. Bland Sutton


ARTICLE II.

### OBSERVATIONS ON THE STRUCTURE AND DEVELOPMENT OF OVARIAN TEETH.

BY T. CHARTERS WHITE AND J. BLAND SUTTON, F. R. C. S.

The subject hitherto had not been investigated in a manner commensurate with its interest, and it was taught by Mr. White and himself that it would be of value to place before the society some facts in connection with this subject. They were unable to state the age at which these teeth developed, for they found children of five, seven, and ten years of age having dermoids with teeth fully erupted. Quite recently they had the opportunity of observing an ovarian in a girl aged seven, and this contained teeth well developed with fangs; the ovarian also contained a lock of hair thirty inches in length. Dermoid cysts in adults and old women would frequently be found, on examination, to contain no teeth. They are equally unable to state the age at which ovarian teeth are shed. Now they knew that the hair in ovaries becomes grey, and the cysts become literally bald, but an edentulous dermoid, in the sense that the teeth have been shed, had yet to be demonstrated. The number of teeth in dermoids varied greatly, in many—probably half, perhaps more—none were detected; when present they were frequently overlooked, and so statistics were unreliable. Two, three, or four teeth is a common number, but if they contain twenty they might be described as numerous. Ovarians have been met with containing 300 or 400 teeth, one on the table contained 350 teeth, a large multilocular dermoid. The structure of these teeth had not previously been carefully described. Only two papers of any importance had been written, one was by Mr. Salter, published in *Guy's Hospital Report*, 1863, and the other by Mr. Coleman, published in the *Transactions* of this Society. But neither went into the matter with any thoroughness. Now, in so far as these ovarian teeth consist of enamel and dentine they correspond with ordinary teeth. They are lodged in bony sockets, and these are lined with periosteum. The bones in which they are lodged are developed afterwards, because in many instances such teeth are lodged in the soft tissues of the tumour; sometimes they are embedded in a piece of bone and look like so many pieces of nails projecting from a piece of wood; and then again, in their early stages they are unassociated with any cartilage. They really correspond to the alveolar process of the jaw, and this was a matter of some importance, because it had given rise to the theory that they are instances of a retained fœtus; the skin and the hair attaching to them gives apparent support to the theory. The fact of the bone being developed round the teeth makes them look very like the alveolus of the upper jaw, and it was perfectly wrong to describe them as due to a retained fœtus. These teeth agree with normal teeth, inasmuch as they consist of enamel and dentine, that is to say, they would find a body of teeth all formed of dentine radiating regularly enough so far as the crown of the teeth is concerned from a central pulp chamber. In the multi-cuspidate teeth they had this regular dentine, and on the top of that a lot of rough hummocky enamel. The other forms of teeth are like bicuspids. There was a remarkable relation of the enamel to the normal enamel on definite pulp chamber; between the dentine and the enamel, there are the usual inter-globular spaces such as are seen in the normal teeth. Attention should also be drawn to the fact that in some of the teeth these hummocks of enamel have very deep clefts, or ravines between them, which in a later stage are filled up with fibrous tissue. In the case of the enamel, the fibres run in all sorts of directions, and although enamel, dentine, and cementum would be found in apparent normal relation, there was in reality the utmost disorder. The amount of cementum is variable, in many teeth it is absent, in some teeth it would be found coating only one side of the fang, but the dentine and enamel are constant. In all these teeth there was only one fang; so far as Mr. Sutton was aware no observer had seen an ovarian tooth with more than one fang. In the cuspidate teeth there was what botanists would call a "tap root." The bicuspids, incisors, and canines had much longer fangs than the multicuspidate teeth. When the crown is long, the fang is short, and inversely when the fang is long, the crown is short. What was more remarkable, these teeth had no pulp-chamber: this was noticed by Salter and also by Coleman. In the multicuspidate teeth sometimes pulp would be found, and sometimes not; in some the pulp is ossified. In some specimens the whole of the pulp is replaced by fat globules and blood. The existence of nerves in the pulp has been demonstrated by Salter, in the paper of 1863 referred to, and Mr. White and Mr. Sutton had been at considerable pains to see if they could get some nerves in pulps, for

Salter's observations had never been confirmed, and as yet they had met with no trustworthy specimens. Quite lately two specimens had come into their possession which seemed to give some indications in support of Salter's view, but their appearance under the microscope was too uncertain to warrant any definite conclusions and so for the present that part of the investigation required more work, though ultimately they expected to be able to demonstrate the presence of nerves. That nerves do exist in dermoids was beyond question, because one of the authors of the paper had had opportunities of experimentally showing this; for instance, the patient in these cases is often able to localize the pricks of a pin with the same certainty as in other parts of the body. Furthermore, there is a peculiarity in the character of the pain arising from dermoids, so that it had been possible to suggest the nature of the tumour from the description of the pain.

So much for the structure; now for the development. So far as Mr. Sutton was aware, no previous observers had described, or figured, or given any satisfactory evidence, as to the modes in which these teeth are developed. Recently Mr. White and himself had been able to trace their development. In dermoids, bodies composed of cells named epithelial pearls are found. The cells are large, but become compressed as they approach the peripheral part of the pearl, and ultimately become lost in the environing capsule. After their enclosure such pearls may increase in size from the growth of the enclosed cells. The epithelial pearls are worthy of close study, for they are allied to enamel organs, and indeed may be regarded as enamel organs, for in one of the illustrations one of them might be seen descending upon a dental papilla beneath like an avalanche. One involved the head of a papilla like a normal enamel germ. These germs lack a definite follicular wall which might possibly explain the deficiency and occasional absence of cementum to the fang. As yet they had not succeeded in detecting any evidence of germs of secondary teeth. It is remarkable that these pearls may remain cellular or give rise to enamel. The relationship of epithelial pearls is of interest in another direction; the pearls had been most closely studied in the median line of the hard and soft palate, as a rule they are small in size but occasionally act as tumour germs, and give rise to neoplasms known as palatine adenomata. It had recently been pointed out that such pearls are occasionally associated with papillæ in the meso-palatine suture, and gave rise to a not uncommon phenomenon, viz., a meso-palatine tooth. In conclusion, much remained to be investigated in ovarian teeth, especially with regard to their character when growing in the ovaries of other mammals.

Mr. Charters White, commenting upon the microscopic specimens which he had prepared, said the forms which had been supplied to him might be assigned to the molars and bicuspids, but were not of the form of normally developed teeth. The molars were stunted in their growth and finished abruptly at their necks in some cases, while in others the root extended as a single tap root brought suddenly to a close. The most striking feature about the molars was their well pronounced and numerous cusps separated from each other by deep fissures. The bicuspids might in many instances have been taken for multiform, canines, the apical foramina in some cases were quite absent. The enamel presented a sodden appearance and was perforated with holes resembling those met with in any worm-eaten wood. On cutting sections with a dental engine and diamond disc or wet piercing saw, the perforations before noticed on the enamel entered and traversed the enamel in tortuous tubes, as if bored by some annelides. The enamel prisms presented every degree of granularity, and were frequently marked by those cross striations usually found in imperfectly developed enamel. The most marked irregularity was perhaps observed in the dentine; the dentinal tubuli were there, but seem to have followed no definite course, presenting a most turbulent appearance. The cementum had been small in quantity in the specimens, and presented no features for special notice. The extraordinary absence of pulp cavity in many teeth was worthy of remark: in all but two cases the pulp was wanting, and in those it was very fragmentary. In conclusion it would be a waste of time to describe irregularities which were not constant.

DISCUSSION.

Mr. Arthur Underwood had come with a very open mind, but still he thought it would be unfair to so able and carefully prepared a paper to allow it to pass without at least some kind of attempted comment. In the first place it would be generally recognized that Mr. Bland Sutton and Mr. Charters White had thrown a great deal of fresh light upon their subject, and he was of opinion that their paper would form a classic. Recently he came upon a case published in a Boston paper, it was a careful description of teeth lodged in two bones which resembled the parietal bones separated from each other by a suture. The teeth were called plainly molars and bicuspids, but they did not seem to Mr. Underwood to be anything of this plain description. He would be happy to give Mr. Bland Sutton the reference to the case if he desired. His next point was in connection with Mr. Sutton's remark that he did not believe there was any recorded instance of ovarian teeth with more than one fang; well, it was a dangerous thing to suggest that Mr. Sutton was possibly in error, but it seemed to Mr. Underwood that the specimen No. 994, which Mr. Sutton had passed round, had 2 fangs at the top; possibly if the bone were chipped away it might prove not to be so. Another point was a reference to epithelial pearls, possibly Mr. Bland Sutton was not aware of some interesting researches from the pen of a Frenchman who discussed these formations at great length, and endeavored to prove that at times they were abnormal teeth, and at other times they were tumours. That reference he would also be pleased to give Mr. Sutton. With regard to the disorder of the tissues, it was obviously present, and he thought such disorder was always present in an abortive attempt to form a tooth. It was present when the pulp forgets to do its natural work and endeavors to form secondary dentine. When there were secondary teeth made they were always of a disordered kind. Of course, Mr. Bland Sutton was careful to cut the particular section illustrated through the centre of the tooth, though it looked from the drawing as if it might be a little off the the pulp. He would be glad if Mr. Sutton would put them in the way of obtaining such specimens as were exhibited. He (Mr. Underwood) would for his part promise to cut them, examine them, and report upon them. In conclusion he thought the society would be able to do greater justice to the paper later on.

Mr. Charters White said he had been careful to cut the specimen as near the median line as possible with a diamond disc and a dental engine. He must say that he very much regretted that he was unable to get more than two sections out of one tooth, therefore he cut each tooth into two sections, and he ground that down pretty flat and polished it so that he thought that they had obtained one side of the median line. He added, at present transverse sections had not been obtained.

Mr. Storer Bennett thought the society was to be congratulated upon the extremely able paper to which they had listened. And as this was the first occasion that Mr. Bland Sutton had been out after a severe illness, they would take it as a very great compliment that he had come down to deliver the paper himself. Mr. Bennett was exceedingly interested in hearing Mr. Sutton's remarks as to enamel being deficient on the crowns of teeth reminding them of the fissures that were to be seen in molars and bicuspids. Mr. Sutton said that at the bottom of these fissures there was fibrous tissue. Now on the authority of Charles Tomes it was known that they had Nasmyth's membrane, and it was very remarkable, as Mr. Sutton had pointed out, that cementum was either not present or was exceedingly thin; that instead of cementum on the Nasmyth's membrane there should be fibrous tissue, Mr. Bennett wished to ask whether he was correct in inferring that Mr. Sutton had found no trace of the membrane passing across the teeth.

Mr. George Cunningham (Cambridge) simply rose for the purpose of asking a question, viz., whether any caries had been found in these cysts. His reason for asking the question was, that at Buda Pesth he saw a cyst of the same nature containing carious teeth. His impression, from noticing Mr. Bland Sutton's specimen, was that caries was present in the various kinds of teeth.

Mr. Charters White, a casual observer might very often mistake some of the foramina for caries. They were brown and corroded, no polish at all on the surface, and they seemed to be eaten into by holes, more like worm holes than anything else, but with perfectly defined borders.

Mr. W. A. Maggs asked for information as to the condition of the multilocular cysts. And with regard to the multicuspidate teeth that seemed altogether exceptional, could Mr. Sutton give any more facts about them?

Mr. F. J. Bennett said that Mr. Bland Sutton's paper induced speculation in the absence of any apparent rational cause for certain departure from the usual course. With reference to calcification, Mr. Sutton had told them that there was no pulp cavity in the teeth from which the section was taken. If they looked at the secondary calcification nodules, which took place in the pulp chambers in old teeth, it seemed to begin in the middle and extend outwards. Might not these teeth, which were so eccentric in many ways, develop calcification in a precisely opposite manner? *i. e.*, commencing outwards and working towards the centre. It seemed quite possible, indeed more probable, as Mr. Sutton had said that in many of the teeth there was no cementum, or only very rudimentary indeed.

Mr. Bennett remarked that in Richard Owen's "Odontology," published many years ago, there was a fine litho of an ovarian tooth, and in it there was no pulp cavity.

Mr. Bland Sutton replying said he was extremely indebted to Mr. Arthur Underwood, and should be glad to be favored with the references to the papers he mentioned. He might say, as to the tooth having a bifid fang, that upon looking at it again he thought Mr. Underwood was probably right. He (Mr. Sutton) had examined many hundreds of these teeth from bifurcated roots without finding them, that at last he supposed he must have got careless. But it was a point that he would take the earliest opportunity of putting beyond dispute. Should Mr. Underwood's view be correct, it would only modify the paper to the extent of stating that bifurcated roots were "of the greatest rarity," instead of that "they were unknown." Now with reference to caries, although he had intentionally excluded the subject in delivering the paper, it had in reality been included in the paper which had been prepared by Mr. Charters White and himself; that caries occurred in ovarian teeth was an assertion which would not hold water for a moment. These ravines in teeth always contained fibrous tissue. Caries in the teeth contained in ovarian dermoids in the sense employed for mouth teeth had yet to be demonstrated. With reference to Mr. F. J. Bennett's remarks, it was a curious thing that both Coleman and Salter had noticed that dentine radiates from the line rather than the pulp chamber. However, it showed the value of bringing a question of that sort before a society instead of writing the subject up in one's study, and packing it off to a journal. In thrashing a subject out before a society many valuable suggestions were often made in the course of discussion. With reference to Mr. Maggs' question, he would say that any number of teeth over twenty or fifty in these cysts was most rare. The dermoid before them was most exceptional. There was nothing peculiar about the teeth, but it would not be wise to draw any conclusion from them. In conclusion, he wished to say that the discussion had been a most profitable one to him.

The President said it afforded him a great deal of pleasure to offer the thanks of the Society to Mr. Bland Sutton and Mr. Charters White for their most interesting, able, and suggestive paper. They were all greatly rejoiced to see Mr. Sutton restored to health again, for had the illness been fatal it would have been a loss, not only to their society, but to the whole scientific world. The next meeting would be on the 14th April, when they would have a contribution from Mr. Henry Sewell on some points in the etiology and pathology of dental caries, illustrated by photo-micrographs of the tissues shown upon a screen by Mr. Andrew Pringle. There would also be a casual communication from Mr. Scott Thomson on splicing engine cords.—*Proceedings of Odontological Society of Great Britain.—London Dental Record.*

